# C188‐9 reduces TGF‐β1‐induced fibroblast activation and alleviates ISO‐induced cardiac fibrosis in mice

**DOI:** 10.1002/2211-5463.13212

**Published:** 2021-06-17

**Authors:** Jiao Liu, Yuxuan Jin, Bei Wang, Jinying Zhang, Shengkai Zuo

**Affiliations:** ^1^ Department of Cardiology First Affiliated Hospital of Zhengzhou University China; ^2^ Key Laboratory of Cardiac Injury and Repair of Henan Province Zhengzhou China; ^3^ Department of Pharmacology and Tianjin Key Laboratory of Inflammation Biology School of Basic Medical Sciences Tianjin Medical University China

**Keywords:** C188‐9, cardiac fibroblasts, cardiac fibrosis, STAT3

## Abstract

Cardiac fibrosis is the final event of heart failure and is associated with almost all forms of cardiovascular disease. Cardiac fibroblasts (CFs), a major cell type in the heart, are responsible for regulating normal myocardial function and maintaining extracellular matrix homeostasis in adverse myocardial remodeling. In this study, we found that C188‐9, a small‐molecule inhibitor of signal transducer and activator of transcription 3 (STAT3), exhibited an antifibrotic function, both *in vitro* and *in vivo*. C188‐9 decreased transforming growth factor‐β1‐induced CF activation and fibrotic gene expression. Moreover, C188‐9 treatment alleviated heart injury and cardiac fibrosis in an isoproterenol‐induced mouse model by suppressing STAT3 phosphorylation and activation. These findings may help us better understand the role of C188‐9 in cardiac fibrosis and facilitate the development of new treatments for cardiac fibrosis and other cardiovascular diseases.

## Graphical abstract

Cardiac fibroblasts (CFs) play important roles in myocardial remodeling by maintaining extracellular matrix homeostasis. Here, we report that C188‐9 exerts an antifibrotic role by inhibiting the phosphorylation and activation of signal transducer and activator of transcription 3 in CFs, both *in vitro* and *in vivo*. C188‐9 may be a potential agent for the clinical therapy of cardiac fibrosis and related heart diseases.

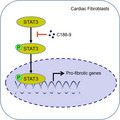

**Abbreviations**
CFs: cardiac fibroblasts
CVDs: cardiovascular diseases
ECM: extracellular matrix
ISO: isoproterenol
STAT3: signal transducer and activator of transcription 3
TGF‐β1: transforming growth factor‐β1
WGA: wheat germ agglutinin

Cardiovascular diseases (CVDs) are the most common disease worldwide, with the highest morbidity and mortality rates [1]. Nearly all forms of heart disease exhibit cardiac fibrosis, characterized by excessive deposition of extracellular matrix (ECM) in the myocardium [2]. Cardiac fibrosis is a repair process that protects the heart from rupture during early stages of injury; however, excessive cardiac fibrosis at the late stage leads to cardiac dysfunction and an increased risk of heart failure [3]. Despite many improvements in treatment strategies, understanding cardiac fibrosis pathogenesis and identifying novel potential therapeutic targets remain an urgent need for heart disease.

An essential cell type in the heart, cardiac fibroblasts (CFs) are the major cells implicated during cardiac fibrosis because they synthesize ECM throughout the myocardium [4, 5]. Quiescent CFs activate and migrate to damaged areas of the heart in response to various damages or stresses and convert into myofibroblasts, which secrete ECM such as collagen and α‐smooth muscle actin (α‐SMA) to maintain myocardium structural and functional integrity [6]. However, CFs may continuously activate, synthesize, and excrete excessive ECM after the initial wound healing process, resulting in the development of cardiac fibrosis [7], and even heart failure [8, 9]. Therefore, clarifying the activation mechanism of CFs and finding agents to inhibit CFs activation may provide new strategies for treating myocardial fibrosis and heart failure.

Indeed, activated CF inhibition and reversion have been proposed as potential therapeutic strategies for cardiac fibrosis [10]. Extensive evidence has demonstrated that signal transducer and activator of transcription 3 (STAT3) signaling participates in CF activation during cardiac fibrosis [10, 11, 12, 13]. C188‐9, a synthetic small‐molecule inhibitor of STAT3, functions by targeting the Src homology 2 (SH2) domain to block phosphorylation and activation [14, 15]. C188‐9 is potent in the treatment of various tumors, such as acute myeloid leukemia and hepatocellular carcinoma [15, 16]. Interestingly, recent studies have indicated that C188‐9 attenuates bleomycin‐induced pulmonary fibrosis [17], skin fibrosis [18], and bile duct ligation‐induced liver fibrosis in mice [19]. However, whether C188‐9 regulates CF activation and subsequent cardiac fibrosis remains to be determined.

This study aimed to investigate the effects of C188‐9 on CF activation and its role in a mouse cardiac fibrosis model. We found that C188‐9 inhibited STAT3 signaling and thus alleviated CF activation, suppressing cardiac fibrosis progression. Our results suggest that these effects of C188‐9 administration protected mice from isoproterenol (ISO)‐induced cardiac dysfunction. Based on these findings, we speculated that C188‐9 is a promising agent for the clinical therapy of cardiac fibrosis and related heart diseases.

## Materials and methods

### Animal experiments

Male C57BL/6 mice aged 10 weeks were purchased from Vital Laboratory Animal Technology Co. Ltd. (Beijing, China) and raised in a pathogen‐free animal house. To cause cardiac dysfunction through the induction of fibrosis, mice were administered ISO (Sigma, St. Louis, MO, USA, 20 mg·kg^−1^·day^−1^) perfusion using osmotic minipumps for 21 days. Mice were then euthanized to harvest their hearts for further analysis. Mice were also intraperitoneally injected with 50 mg·kg^−1^·day^−1^ of C188‐9 (Selleck Chemicals, Houston, TX, USA) for 21 consecutive days, whereas a control group received vehicle (DMSO). All animal experimental protocols and procedures were approved by the Ethics Committee of Tianjin Medical University.

### Isolation and culture of primary cardiac fibroblasts

Primary CFs were isolated and cultured as previously reported [20] with a few modifications. Briefly, male C57BL/6 mice were anesthetized and their hearts collected. After washing with ice‐cold PBS, mouse hearts were minced and digested with type 2 collagenase (Worthington Biochemical Corp., Lakewood, NJ, USA, 0.1%) and trypsin/EDTA (Gibco, Grand Island, NY, USA, 0.1%). Heart cells were centrifuged at 1000 ***g*** for 5 min, suspended in Dulbecco's Modified Eagle's medium (DMEM; Gibco, Grand Island, NY, USA) containing 10% FBS (Gibco, Grand Island, NY, USA), and plated in culture dishes. After 2 h, nonadherent cells were removed. Adherent cells were CFs and detected via immunofluorescence using vimentin staining. After two or four passages, CFs were used for subsequent experiments.

### Cell culture and treatment

Primary CFs were cultured in DMEM supplemented with 10% (v/v) FBS, 100 U·mL^−1^ penicillin, and 100 U·mL^−1^ streptomycin in a humidified incubator at 37 °C and 5% (v/v) CO_2_. To induce fibrosis, cells were stimulated with transforming growth factor‐β1 (TGF‐β1; R and D Systems, Minneapolis, MN, USA, 10 ng·mL^−1^) as previously reported [21]. C188‐9 (10 μm) or vehicle control was administered to CFs 12 h before TGF‐β1 treatment.

### Quantitative reverse transcription‐PCR

Total RNA from cultured cells or mouse tissues was extracted using TRIzol Reagent (Thermo Fisher Scientific, Waltham, MA, USA), and 1 μg of RNA was reverse transcribed to cDNA using the PrimeScript™ RT Reagent Kit (Takara, Japan), following manufacturer's protocol. The cDNA was then used for PCR amplification with gene‐specific primers. *GAPDH* was the endogenous control. The primers used were as follows: *Col1a1*, F 5′‐GCTCCTCTTAGGGGCCACT‐3′, R 5′‐CCACGTCTCACCATTGGGG‐3′; *Col1a2*, F 5′‐GTAACTTCGTGCCTAGCAACA‐3′, R 5′‐CCTTTGTCAGAATACTGAGCAGC‐3′; *Col3a1*, F 5′‐CCTGGCTCAAATGGCTCAC‐3′, R 5′‐CAGGACTGCCGTTATTCCCG‐3′; *α‐SMA*, F 5′‐GTCCCAGACATCAGGGAGTAA‐3′, R 5′‐TCGGATACTTCAGCGTCAGGA‐3′; *ANF*, F 5′‐GCTTCCAGGCCATATTGGAG‐3′, R 5′‐GGGGGCATGACCTCATCTT‐3′; *BNP*, F 5′‐GAGGTCACTCCTATCCTCTGG‐3′, R 5′‐GCCATTTCCTCCGACTTTTCTC‐3′; *GAPDH*, F 5′‐AGGTCGGTGTGAACGGATTTG‐3′, R 5′‐TGTAGACCATGTAGTTGAGGTCA‐3′.

### Western blotting

Total protein was extracted from cultured cells or mouse hearts using RIPA lysis buffer (Beyotime, Shanghai, China) supplemented with proteinase inhibitor cocktail (Roche Molecular Biochemicals, Mannheim, Germany) as previously described [22]. Protein concentration was measured using a BCA protein assay kit (Thermo Fisher Scientific, Waltham, MA, USA). Proteins were then separated via SDS/PAGE (10%) and electrotransferred to polyvinylidene difluoride membranes (Millipore, Billerica, MA, USA), incubated with the indicated antibodies, and developed using chemiluminescence methods. The following antibodies were used as follows: anticollagen I (1 : 1000; catalog number: 14695‐1‐AP; ProteinTech Group, Rosemont, IL, USA), anti‐α‐SMA (1 : 1000; catalog number: 14395‐1‐AP; ProteinTech Group, Rosemont, IL, USA), anti‐STAT3 (1 : 1000; catalog number: 9139; Cell Signaling Technology, Danvers, MA, USA), anti‐p‐STAT3 (1 : 1000; catalog number: 9145; Cell Signaling Technology, Danvers, MA, USA), and anti‐GAPDH (1 : 10 000; catalog number: 60004‐1‐Ig; ProteinTech Group, Rosemont, IL, USA).

### Echocardiography

Echocardiography and cardiac function evaluation were performed as previously described [23].

### Assessment of cardiac fibrosis

Mouse hearts were harvested, fixed with paraformaldehyde, embedded in paraffin, and sectioned at 5 µm. Tissue slices were subjected to Masson's trichrome staining (Sigma) and wheat germ agglutinin (WGA; Thermo Fisher Scientific) staining, following manufacturer protocol. Average collagen area was measured using ImageJ‐v1.8.0, as described previously [24]. Briefly, Masson's trichrome‐stained sections were captured with a BX51 microscope (Olympus, Tokyo, Japan), and collagen area (blue) was measured with the “image‐color” plugin for imagej (NIH, Bethesda, MD, USA).

### Statistics

All quantitative data were tested using the Shapiro–Wilk normality test through SPSS version 21.0 (IBM Inc, Armonk, NY, USA) and presented as mean ± standard error of the mean (SEM). The normality assumption was not violated (*P* > 0.05). Statistical differences between groups were calculated with two‐way ANOVA, followed by Tukey's *post hoc* test. Significance was set at *P* < 0.05. Statistics were performed using Microsoft Excel and prism 7.0 (GraphPad Software, San Diego, CA, USA).

## Results

### C188‐9 reduces fibroblast activation in CFs

**Fig. 1:**
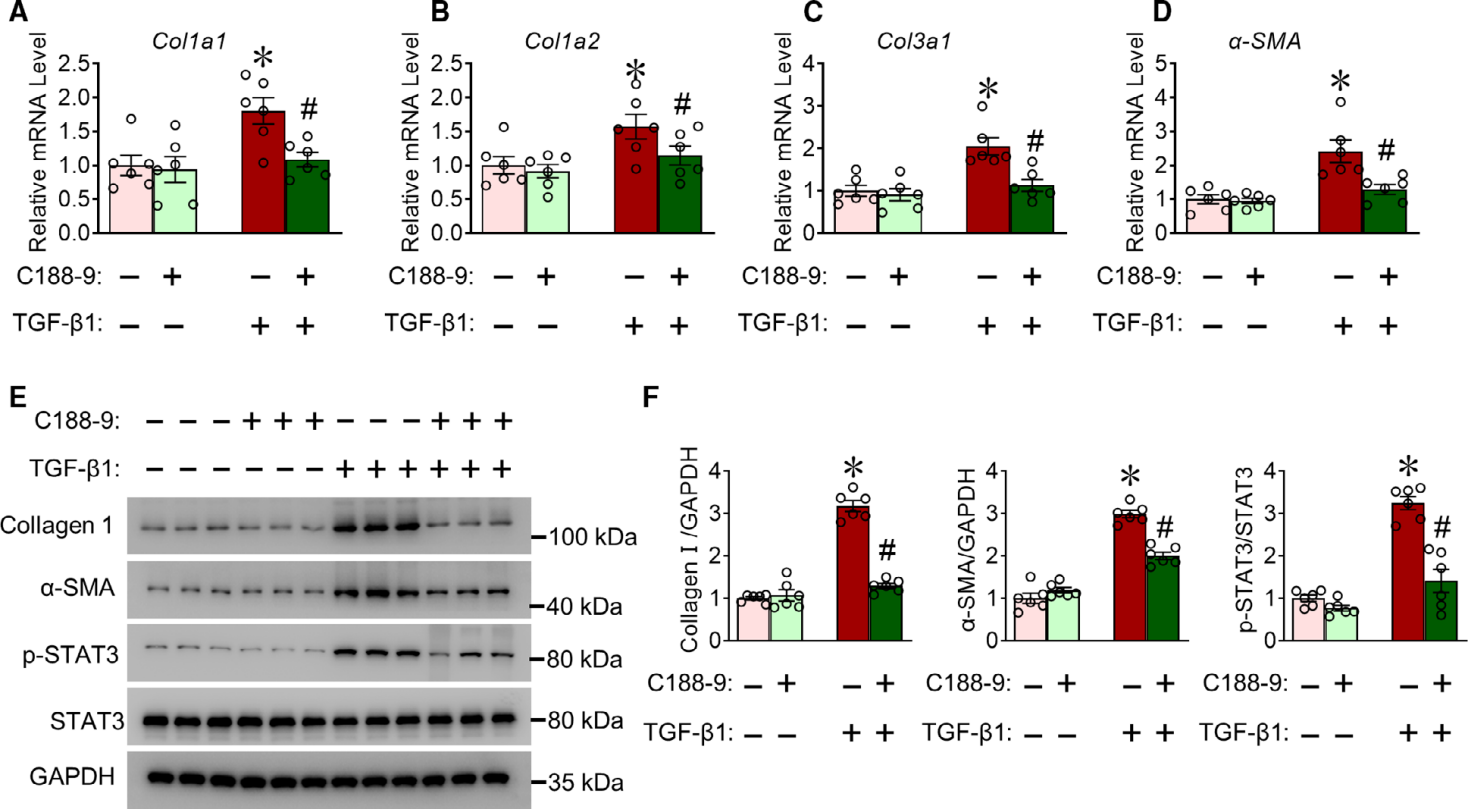
C188‐9 reduces TGF‐β1‐induced fibroblast activation. CFs were isolated, pretreated with C188‐9 (10 μm) for 12 h, and then stimulated with C188‐9 and TGF‐β1 for another 48 h. Total RNA or protein extracts were isolated. (A–D) Quantitative RT‐PCR (qRT‐PCR) of *Col1a1* (A), *Col1a2* (B), *Col3a1* (C), and *α‐SMA* (D) transcript levels in CFs. Results are normalized to 1, *n* = 6, *P < 0.05, TGF‐β1‐DMSO vs. vehicle DMSO. ^#^ *P* < 0.05, TGF‐β1‐C188‐9 vs. TGFβ1‐DMSO; two‐way ANOVA, followed by Tukey's *post hoc* test. (E) Western blots of collagen Ⅰ, α‐SMA, p‐STAT3, and total STAT3 levels. GAPDH was used as loading control. (F) Quantification of relative collagen I and α‐SMA levels. *n* = 6. *P < 0.05, TGF‐β1‐DMSO vs. vehicle DMSO. ^#^ *P* < 0.05, TGF‐β1‐C188‐9 vs. TGFβ1‐DMSO; two‐way ANOVA, followed by Tukey's *post hoc* test. Data are presented as mean ± SEM.

We first employed CFs to investigate whether C188‐9 has any effect on cardiac fibrosis. We isolated CFs from C57BL/6 mouse hearts and pretreated them with C188‐9 or vehicle control, followed by TGF‐β1 stimulation to induce fibrosis. After isolating total RNA, quantitative reverse transcription (qRT)‐PCR detected mRNA levels of fibrosis markers. As expected, TGF‐β1 stimulation markedly increased mRNA expression of Col1a1 (Fig. 1A), Col1a2 (Fig. 1B), Col3a1 (Fig. 1C), and α‐SMA (Fig. 1D), whereas C188‐9 treatment significantly reduced this effect (Fig. 1A–D). In addition, western blot measured type I collagen (collagen I) and α‐SMA proteins. C188‐9 treatment also decreased TGF‐β1‐induced expression of collagen I, α‐SMA, and STAT3 phosphorylation, consistent with mRNA data (Fig. 1E,F). Collectively, these results indicate that C188‐9 decreased TGF‐β1‐induced fibrotic response in CFs.

### C188‐9 treatment ameliorated ISO‐induced cardiac dysfunction in mice

**Fig. 2:**
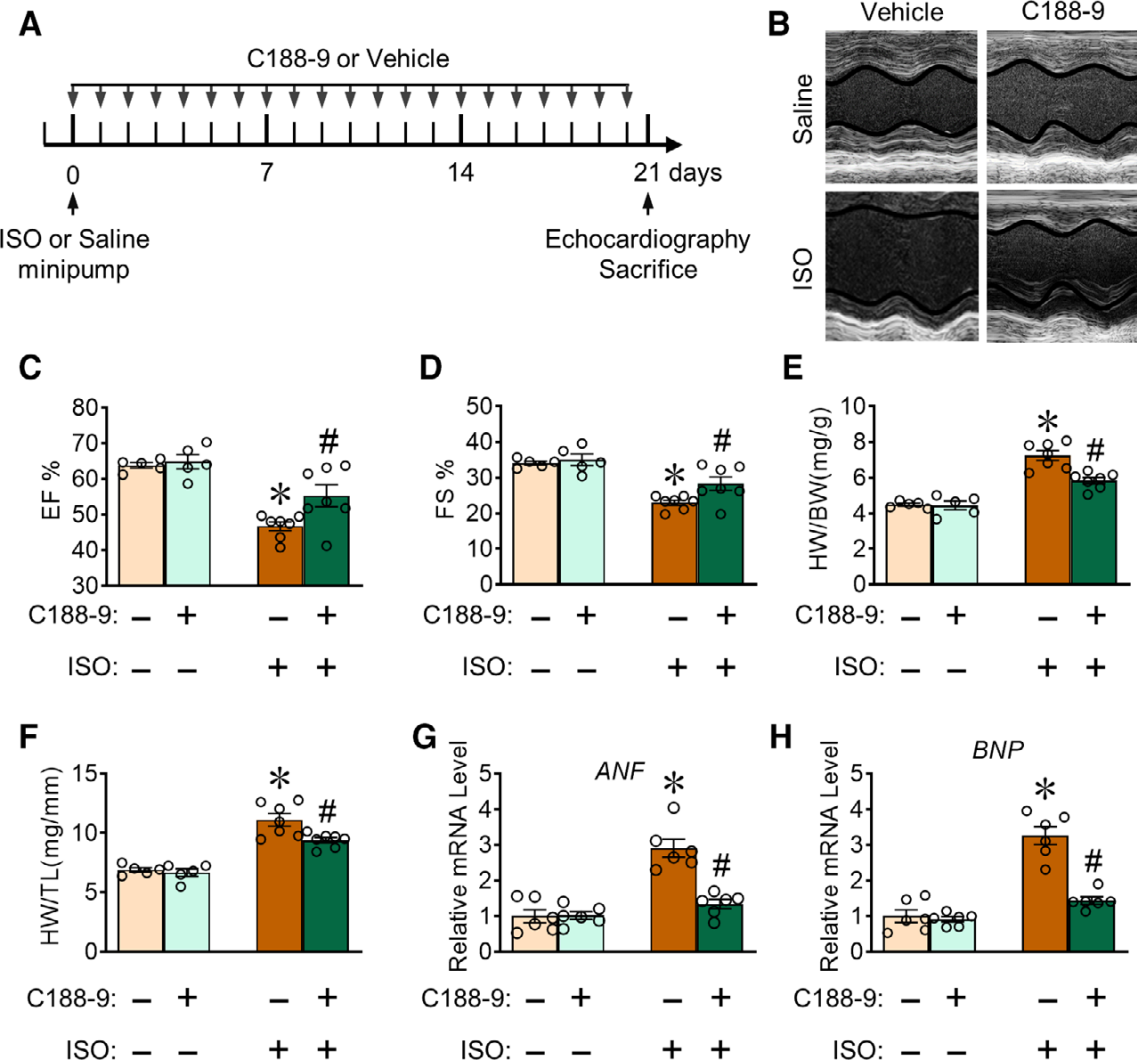
C188‐9 treatment prevents ISO‐induced left ventricular dilatation and dysfunction in mice. C57BL/6 mice aged 10 weeks were administered ISO perfusion with or without C188‐9 treatment. (A) Scheme of C188‐9 treatment in the ISO‐induced mouse model. Mice received ISO perfusion, echocardiography was performed, and heart tissues were collected on day 21. (B) Representative M‐mode echocardiography of the left ventricular chamber. (C, D) Statistical analysis of ejection fraction (EF) (C) and fractional shortening (FS) (D). Saline group, *n* = 5; ISO group, *n* = 7; *P < 0.05, ISO‐vehicle vs. saline‐vehicle. ^#^ *P* < 0.05, ISO‐C188‐9 vs. ISO‐vehicle. Two‐way ANOVA, followed by Tukey's *post hoc* test. (E, F) Heart weight‐to‐body weight ratio (HW/BW) (E) and heart weight‐to‐tibia length ratio (HW/TL) (F) were assessed. Saline group, *n* = 5; ISO group, *n* = 7; *P < 0.05, ISO‐vehicle vs. saline‐vehicle. ^#^ *P* < 0.05, ISO‐C188‐9 vs ISO‐vehicle. Two‐way ANOVA, followed by Tukey's *post hoc* test. (G, H) qRT‐PCR of *ANF* (G) and *BNP* (H) mRNA levels in ISO or/and C188‐9‐treated mouse heart tissues. *n* = 6. *P < 0.05, ISO‐vehicle vs. saline‐vehicle. ^#^ *P* < 0.05, ISO‐C188‐9 vs. ISO‐vehicle. Two‐way ANOVA, followed by Tukey's *post hoc* test. Data are presented as mean ± SEM.

Because CFs play important roles in cardiac function, we examined the effect of C188‐9 treatment on ISO‐induced cardiac dysfunction. We treated 10‐week‐old C57BL/6 mice with ISO perfusion for 3 weeks, while simultaneously injecting C188‐9 treatment or vehicle control. Before ISO challenge and after 21 days, we assessed cardiac function using echocardiography (Fig. 2A). Cardiac function (primarily measured as ejection fraction and fractional shortness) sharply decreased after ISO perfusion, and C188‐9 treatment (50 mg·kg^−1^·day^−1^) exerted a protective effect against cardiac dysfunction in ISO‐induced mice (Fig. 2B–D). After obtaining body weight (BW), mice were euthanized to collect heart tissue and to measure heart weight (HW) and tubular length (TL). After ISO perfusion, HW/BW (Fig. 2E) and HW/TL (Fig. 2F) ratio increased, whereas the C188‐9 group mice exhibited significantly improved indexes compared with vehicle (Fig. 2E,F). Atrial natriuretic factor (ANF) and brain natriuretic peptide (BNP) are widely recognized markers of cardiac damage. Compared with vehicle, we observed markedly downregulated ANF and BNP RNA expression in injured hearts from C188‐9‐treated mice after ISO perfusion (Fig. 2G,H). Taken together, these data demonstrate that C188‐9 treatment alleviates ISO‐induced cardiac dysfunction in mice.

### C188‐9 attenuates ISO‐induced pathological cardiac fibrosis and hypertrophy in mice

**Fig. 3:**
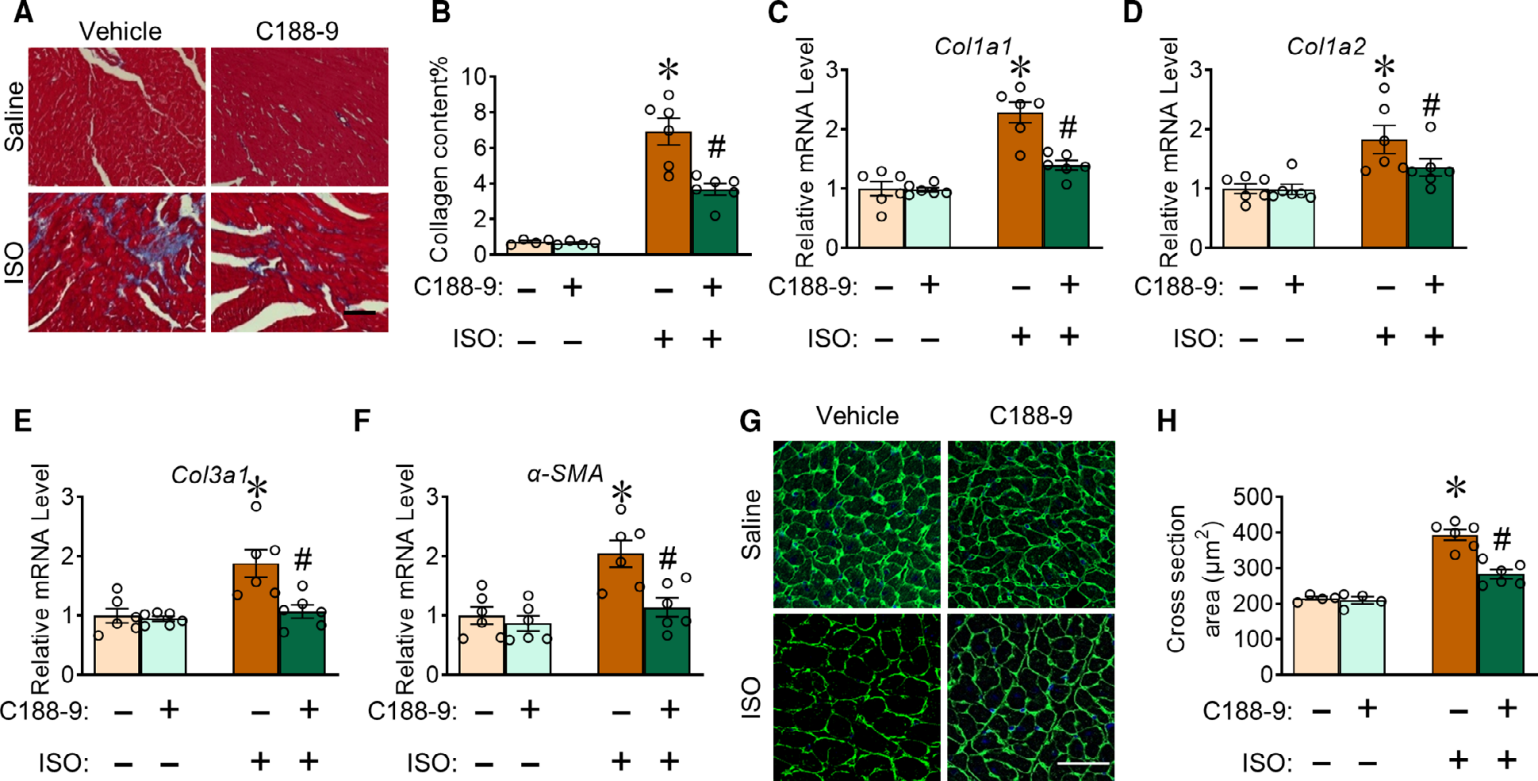
C188‐9 treatment suppresses ISO‐induced fibrosis and hypertrophy in mice. (A, B) Representative images (A) and quantification (B) of Masson's trichrome staining in heart sections from saline‐ or ISO‐treated mice. Saline group, *n* = 4; ISO group, *n* = 6; *P < 0.05, ISO‐vehicle vs. saline‐vehicle. ^#^ *P* < 0.05, ISO‐C188‐9 vs. ISO‐vehicle. Scale bar: 50 μm. (C–F) qRT‐PCR of *Col1a1* (C), *Col1a2* (D), *Col3a1* (E), and *α‐SMA* (F) in mouse heart tissues. *n* = 6. *P < 0.05, ISO‐vehicle vs saline‐vehicle. ^#^ *P* < 0.05, ISO‐C188‐9 vs. ISO‐vehicle. (G, H) Representative images (G) and quantification (H) of WGA staining in heart cross sections from saline‐ or ISO‐treated mice. Saline group, *n* = 4; ISO group, *n* = 6; *P < 0.05, ISO‐vehicle vs. saline‐vehicle. ^#^ *P* < 0.05, ISO‐C188‐9 vs. ISO‐vehicle. Scale bar: 50 μm. Two‐way ANOVA, followed by Tukey's *post hoc* test. Data are presented as mean ± SEM.

Next, we determined whether C188‐9 affected ISO‐induced fibrosis and hypertrophy in mice. C57BL/6 mice were administered ISO perfusion for 3 weeks to stimulate fibrosis, during which C188‐9 or vehicle control was administered daily (Fig. 2A). Masson's trichrome staining was performed to determine fibrosis severity and revealed that C188‐9 treatment markedly reduced ISO‐induced cardiac fibrosis in mice by 21 days after ISO treatment (Fig. 3A,B). Accordingly, the expression levels of Col1a1, Col1a2, Col3a1, and α‐SMA were decreased in heart tissues of C188‐9‐treated mice at days 21 post‐ISO treatment (Fig. 3C–F). Additionally, WGA staining showed a marked reduction in cardiomyocyte size in C188‐9‐treated mice after ISO treatment (Fig. 3G,H). Collectively, these results establish the protective role of C188‐9 against ISO‐induced cardiac fibrosis and hypertrophy in mice.

### C188‐9 treatment attenuated ISO‐induced fibrotic gene expression and STAT3 phosphorylation in mouse heart tissues

**Fig. 4:**
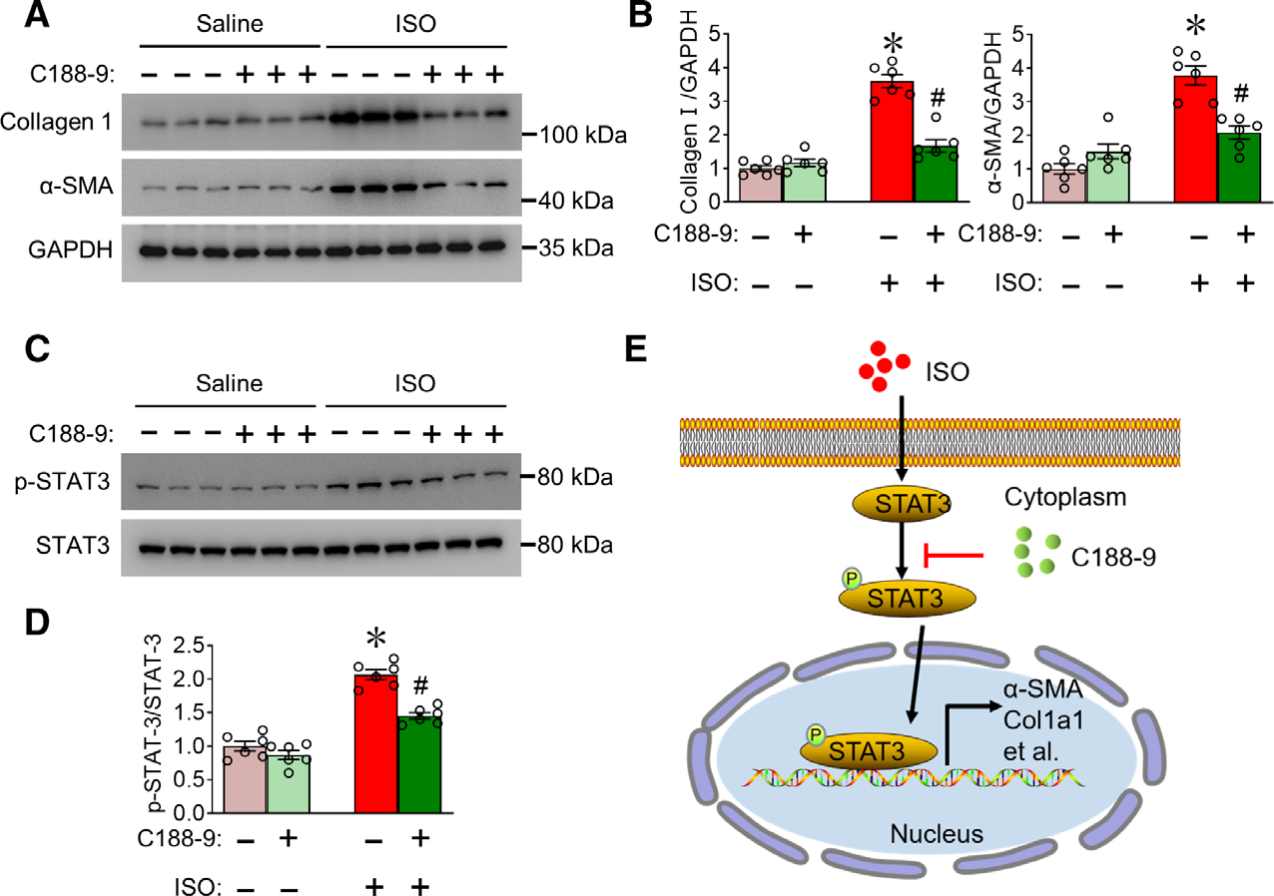
C188‐9 treatment inhibits fibrotic protein expression and STAT3 phosphorylation in cardiac tissues of mice. (A) Western blots of collagen Ⅰ and α‐SMA levels in ISO‐ or saline‐treated mouse heart tissues with or without C188‐9 treatment. GAPDH was used as loading control. (B) Quantification of relative collagen I and α‐SMA levels. *n* = 6 *P < 0.05, ISO‐vehicle vs. saline‐vehicle. ^#^ *P* < 0.05, ISO‐C188‐9 vs. ISO‐vehicle. Two‐way ANOVA, followed by Tukey's *post hoc* test. (C) Western blots of p‐STAT3 and total STAT3 levels in ISO‐ or saline‐treated mouse heart tissues with or without C188‐9 treatment (*n* = 6). (D). Quantification of relative p‐STAT3/STAT3 levels. *n* = 6 *P < 0.05, ISO‐vehicle vs. saline‐vehicle. ^#^ *P* < 0.05, ISO‐C188‐9 vs. ISO‐vehicle. Two‐way ANOVA, followed by Tukey's *post hoc* test. Data are presented as mean ± SEM. (E) Proposed model for C188‐9 regulation of fibrosis‐related gene expression in CFs through STAT3 phosphorylation. Data are presented as mean ± SEM.

Next, we detected protein changes in ISO‐induced cardiac fibrosis with or without C188‐9 treatment. Administration of C188‐9 significantly suppressed collagen I and α‐SMA protein levels in mouse hearts at 21 days post‐ISO perfusion (Fig. 4A,B). The STAT3 pathway plays a critical role in cardiac fibrosis by regulating CF phenotype and activation, studies show that C188‐9 inhibition of STAT3 phosphorylation suppresses skin fibrosis [18, 25], and we tested whether C188‐9 inhibited cardiac fibrosis via STAT3, analyzing ISO‐induced mouse hearts with or without C188‐9 treatment. We found that STAT3 phosphorylation (p‐STAT3) increased in ISO‐treated mice hearts compared with vehicle control, but C188‐9 obviously decreased this increase in p‐STAT3 (Fig. 4C,D). Hence, inhibition of STAT3 signaling is the mechanism underlying the protective role of C188‐9 against ISO‐induced cardiac fibrosis in mice (Fig. 4E).

## Discussion

Activation and ECM production of CFs is a critical process underlying cardiac fibrosis [2]. Here, we demonstrated that a small‐molecule inhibitor of STAT3, C188‐9, blocked CF activation. More importantly, our data revealed that C188‐9 treatment improved cardiac function in mice by suppressing ECM production and attenuating heart fibrosis, thus laying the groundwork for the development of novel drugs to treat CVDs.

TGF‐β signaling is the most extensively studied factor in CF activation, and TGF‐β/Smad is the canonical pathway in pathological cardiac fibrosis [26, 27]. TGF‐β induces ECM protein production depending on Smad3 phosphorylation, as demonstrated in research showing that fibroblast‐specific Smad3 deletion attenuates cardiac fibrotic response and ECM protein expression [28]. In addition to TGF‐β/Smad signaling, an increasing number of studies have found that STAT3 signaling plays essential roles in cardiac physiology and pathology [10, 11, 13, 25]. For example, cardiac constitutive STAT3 activation protects the myocardium from ischemia/reperfusion injury [29]. In a mouse myocardial infarction model, STAT3 inhibition contributed to the reduction in ECM synthesis and protection of cardiac remodeling [30]. Interestingly, TGF‐β1 induces STAT3 phosphorylation and contributes to fibrotic protein accumulation in cultured CFs, whereas genetic or pharmacological blocking of STAT3 activation alleviated cardiac fibrosis by inhibiting autophagy [31]. These observations imply that STAT3 is important in cardiac fibrosis and that inhibiting STAT3 action is a promising strategy in cardiac fibrosis treatment. Here, we found that C188‐9 inhibited STAT3 phosphorylation and ECM expression in an ISO‐induced mouse model. Although we did not explore the mechanistic link between C188‐9 and TGF‐β, our data support the notion that STAT3 signaling is required for regulating cardiac fibrosis.

C188‐9 was recently identified to target the phosphotyrosine peptide‐binding site within the STAT3 SH2 domain, inhibiting granulocyte colony‐stimulating factor‐induced STAT3 phosphorylation and inducing apoptosis in acute myeloid leukemia cell lines [15]. Subsequent research on C188‐9 focused on antitumor potential. C188‐9 treatment inhibited the growth of hepatocellular carcinoma and protected liver function in hepatocyte‐specific‐Pten‐deficient mice [16]. C188‐9 also improves the therapeutic efficacy of 5‐Aza‐2′‐deoxycytidine against pancreatic cancer by regulating demethylation [32]. Other recent studies have shown that C188‐9 is a potential drug for the treatment of fibrotic diseases, decreasing pulmonary fibrosis and reducing collagen deposition in an intraperitoneal bleomycin‐induced mouse model [17]. In other mouse model, C188‐9 decreased TGF‐β‐induced STAT3 phosphorylation and reduced skin fibrosis [18]. Consistent with these findings, our results showed that C188‐9 protects the heart from ISO‐induced cardiac fibrosis. Importantly, C188‐9 is well tolerated and does not cause pathological abnormalities in mice [33]. Despite these promising findings, the clinical application of C188‐9 as a drug requires further investigation.

In summary, our observations demonstrated that C188‐9 attenuated ISO‐induced cardiac fibrosis by inhibiting STAT3 signaling. Thus, C188‐9 is a candidate therapeutic drug for the treatment of cardiac fibrosis.

## Conflict of interest

The authors declare no conflict of interest.

## Author contributions

JL and SZ designed the project, carried out most of the experiments, collected the data, and performed the analysis. BW and YJ helped with the mouse experiment. JL and SZ wrote the manuscript. JZ revised the manuscript.

## Data Availability

The data that support the findings of this study are available from the corresponding author upon reasonable request.
